# Estimation of life expectancy and healthcare cost in rheumatoid arthritis patients with and without depression: a population-based retrospective cohort study

**DOI:** 10.3389/fmed.2023.1221393

**Published:** 2023-11-02

**Authors:** Ying-Ming Chiu, Joung-Liang Lan, Wei-Lieh Huang, Chi-Shin Wu

**Affiliations:** ^1^Department of Allergy, Immunology, and Rheumatology, Tungs’ Taichung MetroHarbor Hospital, Taichung, Taiwan; ^2^Department of Post-Baccalaureate Medicine, College of Medicine, National Chung Hsing University, Taichung, Taiwan; ^3^Department of Nursing, Jen Teh Junior College of Medicine, Nursing and Management, Miaoli, Taiwan; ^4^Rheumatology and Immunology Center, China Medical University Hospital, Taichung, Taiwan; ^5^Department of Medicine, China Medical University, Taichung, Taiwan; ^6^Department of Psychiatry, National Taiwan University Hospital Yunlin Branch, Douliu, Taiwan; ^7^Department of Psychiatry, College of Medicine, National Taiwan University, Taipei, Taiwan; ^8^National Center for Geriatrics and Welfare Research, National Health Research Institutes, Miaoli, Taiwan

**Keywords:** rheumatoid arthritis, depression, healthcare cost, life expectancy, disease burden

## Abstract

**Purpose:**

This study aimed to estimate the lifetime healthcare costs and loss of life expectancy (loss-of-LE) among patients with incident rheumatoid arthritis (RA) with and without depression.

**Methods:**

This 18 years longitudinal cohort study used data from Taiwan’s National Health Insurance Research Database. In total, 43,311 patients with RA were included. Among them, 1,663 patients had depressive disorders in the year preceding the RA diagnosis. The survival function for patients with RA with or without depression was estimated and extrapolated over a lifetime using the rolling extrapolation algorithm. The loss-of-LE was calculated by comparing the sex, age, and calendar year-matched referents from vital statistics. The average monthly cost was calculated as the sum of the monthly costs for all patients divided by the number of surviving patients. Lifetime healthcare costs were estimated by multiplying the monthly average cost by the monthly survival probability.

**Results:**

The loss-of-LE for RA patients with and without depression was 5.60 years and 4.76 years, respectively. The lifetime costs of RA patients with and without depression were USD$ 90,346 and USD$ 92,239, respectively. However, the annual healthcare costs were USD$ 4,123 for RA patients with depression and USD$ 3,812 for RA patients without depression. Regardless of sex or age, RA patients with depression had higher annual healthcare costs than those without depression.

**Conclusion:**

Patients with RA and depression have a high loss-of-LE and high annual healthcare costs. Whether treating depression prolongs life expectancy and reduces healthcare costs warrants further investigation.

## 1. Introduction

Rheumatoid arthritis (RA) is a chronic medical condition characterized by systemic joint inflammation. It has numerous negative health indicators, including pain, disability, low quality of life, and increased mortality (1, 2). In addition, RA is associated with multiple comorbid conditions (3), with depressive disorder being one of the most common. A meta-analysis study showed a depression prevalence of approximately 16.8% among patients with RA (4). Notably, depressive disorders lead to a poor prognosis for many chronic diseases, including diabetes mellitus and cardiovascular diseases (5, 6). Depressive disorder is also an independent risk for mortality (7).

Literature reviews have shown that patients with RA and depression experience more pain, fatigue, and disability than those with RA alone (8, 9). However, few studies have demonstrated that depression is associated with high mortality in RA. One hospital-based cohort study, including 1,290 RA patients with an 18 years observation period, demonstrated that clinical depression was associated with a 2.2-fold increased risk of mortality (10). Another longitudinal study of 882 RA patients with a 14 years follow-up found that depression was associated with 1.35-fold hazard ratios of mortality (11). Moreover, a Finnish case study identified that 10 women and 9 men with RA died by suicide. The authors found that patients who died by suicide had more depressive disorders than the control group, whereas such associations were not found among male patients with RA (12).

The economic burden of RA is substantial (1) and may be increased by comorbid depression. Two studies using the U.S. insurance claim database revealed that RA patients with depression had higher health service utilization during the 12 months post-diagnosis period. This included hospitalization, physician visits, and emergency room visits, thereby having higher all-cause direct healthcare costs than patients with RA alone (13, 14). Another study conducted in Japan also showed that comorbid depression was associated with increased total healthcare utilization and costs (15). However, these studies only estimated healthcare costs in the first 12 months after diagnosis. The increase in lifetime healthcare expenditures due to depression has not yet been explored.

Using a nationwide longitudinal cohort of incident RA patients with an 18 years follow-up period, this study aimed to estimate the lifetime healthcare costs and loss of life expectancy (loss-of-LE) of RA patients with and without depression.

## 2. Materials and methods

### 2.1. Study approval

The study was approved by the Research Ethics Committee of China Medical University and Hospital (IRB number: CMUH108-REC2-119).

### 2.2. Study population and datasets

Taiwan’s National Health Insurance (NHI) was implemented in 1995. Since 2004, more than 99% of Taiwanese citizens have been covered (16). In the NHI Registry for Catastrophic Illness Patients, two physicians confirmed the catastrophic illness diagnosis. First, we identified a retrospective cohort of RA patients from the Registry for Catastrophic Illness Patients between 1999 and 2017 (International Classification of Diseases, Ninth Revision, ICD-9-CM codes 714.0, 714.1, 714.2, and 714.81, and ICD-10-CM codes M05, M06.0, M06.2, M06.3, M06.8, and M06.9). Patients younger than 16 years old at the time of diagnosis were excluded. To ensure data accuracy, an examination was carried out involving patients’ birth year and sex in both the NHI Registry for Catastrophic Illness and the Registry of Beneficiaries. Subsequently, individuals displaying discrepancies or missing information were excluded from the analysis. The survival status of patients with RA was verified by linkage to the National Mortality Registry. All patients with RA were followed until death or 31 December 2017. RA patients with depression were defined as having a diagnosis of depression in the year before the RA diagnosis. According to the ICD diagnosis codes, depression was categorized into major depression (ICD-9-CM codes 296.2, 296.3; ICD-10-CM codes: F32.1–F32.9; F33.1–F33.9) and minor depression (ICD-9-CM code: 300.4, 311; ICD-10-CM code: F34.1). In cases where a patient presented with both major and minor depression, the classification would prioritize major depression. In the analyses of life expectancy (LE) and lifetime costs, the RA cohort was further stratified by depression subtype, sex, and age at RA diagnosis (<50, between 50 and 64, and ≥65 years).

### 2.3. Estimation of LE and loss-of-LE

To estimate the LE of RA patients, we applied the semiparametric survival extrapolation method by Hwang and Wang (17, 18) and mathematically validated it by Fang et al. (19). This method has been used in RA by Chiu et al. (1) and is widely applied in other medical or psychiatric disorders (20, 21). Survival functions were generated by the Kaplan–Meier method until the end of the follow-up. First, a reference cohort was matched to the age, sex, and calendar year at diagnosis of the RA cohort. It was established using the Monte Carlo method based on Taiwan’s National Vital Statistics life table. Second, the survival rates of the RA cohort and the reference cohort were logit-transformed at each time point “*t*.” By fitting a restricted cubic spline model, we could predict the survival rate for the next month. Third, the predicted survival rate for the next month was regarded as the actual observation, and the data used in the previous extrapolation for the first month were ignored. Furthermore, the restricted cubic spline model was refitted again by repeating this step until the survival rate was close to zero. This procedure is called the rolling extrapolation algorithm.

The area under the estimated survival curve of the RA cohort was the LE after RA diagnosis. Moreover, loss-of-LE refers to the area difference between the survival curves of the RA and reference cohorts. We used the open-source R package iSQoL2 to infer the survival curves. The standard error and 95% confidence intervals were estimated through a permutation test. Group differences were assessed using a *z*-test to compute *p*-values.

To facilitate comparisons with previous studies, we conducted a Cox regression model to estimate the hazard ratios of mortality while adjusting for age, sex, and calendar year at diagnosis.

### 2.4. Estimation of lifetime healthcare expenditures for patients

We collected reimbursement data from the NHI database to estimate lifetime healthcare costs for patients with RA. First, monthly patient costs were summed and divided by each month’s surviving cases to calculate the average monthly cost. Assuming that healthcare costs would increase in the months near the end of life, the average cost function was estimated by weighing the patient’s average cost over the previous months. The lifetime healthcare costs were obtained by multiplying the monthly average cost to the survival probability (18). We adjusted annual NHI costs based on the Consumer Price Index (CPI) to account for inflation. For consistency, the extrapolated costs were adjusted at a discount rate of 3% per year. Currency values were expressed in 2017 US dollars (1 USD = 30.44 TWD).

### 2.5. Sensitivity analysis

Given that biological therapies were introduced in 2003 in Taiwan, healthcare costs increased dramatically. The sensitivity analysis included only patients diagnosed between 2003 and 2017.

## 3. Results

### 3.1. Patient characteristics

There were 43,311 new cases of RA from 1999 to 2017, of which 1,662 (3.8%) had a diagnosis of depression prior to the RA diagnosis. Among the 33,225 female patients with RA, 1,383 (4.2%) had depressive disorders. Among the 10,086 male patients with RA, 279 (2.8%) had depressive disorders.

### 3.2. Estimation of LE and loss-of-LE

**Figure 1:**
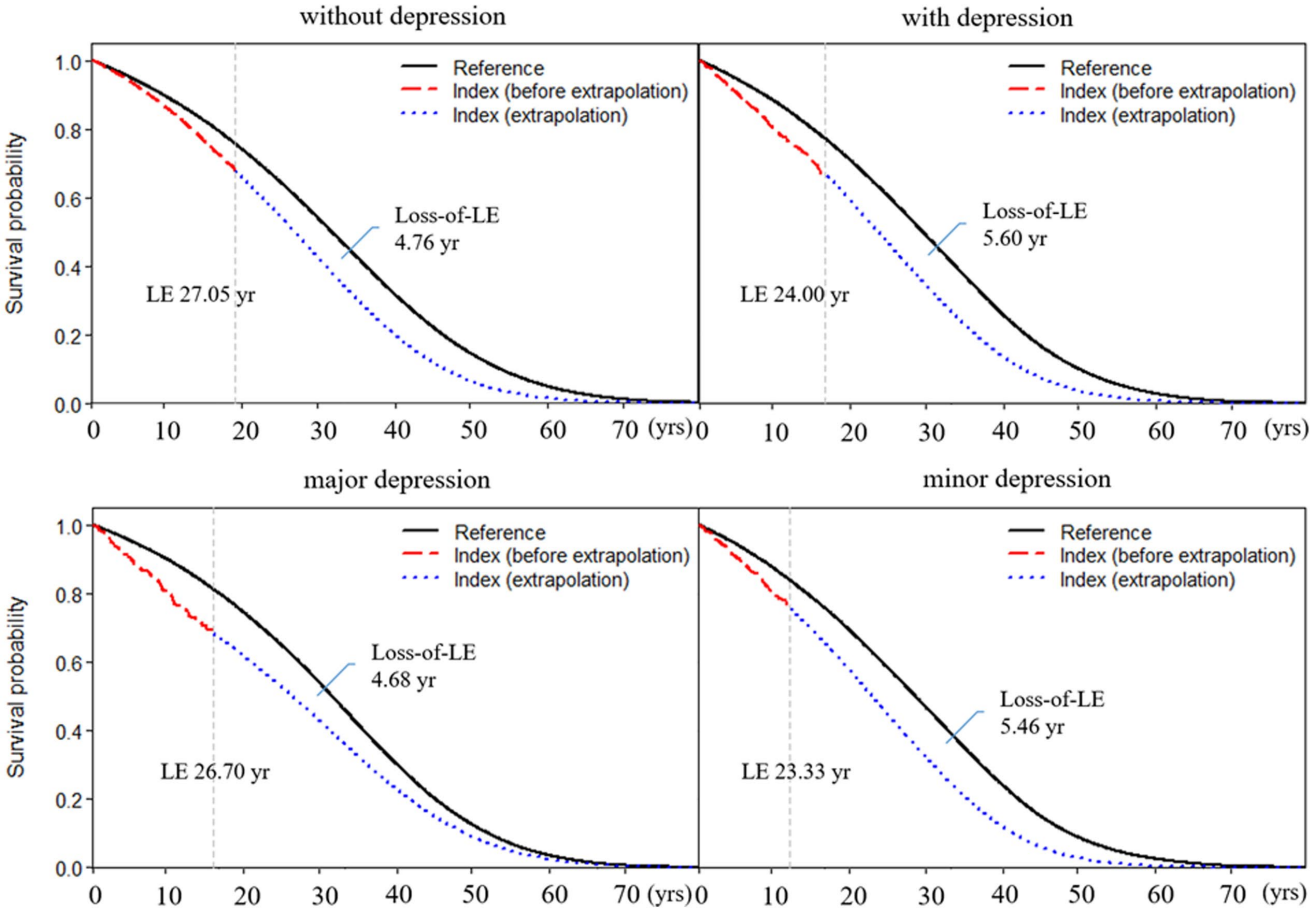
Life expectancy and loss of life expectancy after diagnosis of rheumatoid arthritis stratified by depression. The red line indicates the survival curve of true data from rheumatoid arthritis patients, and the blue line indicates the survival curve of extrapolation from rheumatoid arthritis. The black line indicates the survival curve of the sex-, age-, and calendar year-matched reference cohort.

The average age at diagnosis of patients with RA without depression was 52.68 years old, the mean LE after diagnosis was 27.05 years, and the loss-of-LE was 4.76 years (95% CI: 3.63–5.72). In contrast, the average age at diagnosis of RA patients with depression was 55.41 years old, the mean LE after diagnosis was 24.00 years, and the loss-of-LE was 5.60 years (1.99–8.92). While the 95% confidence intervals for loss-of-LE overlapped between RA patients with and without depression (*p*-value = 0.667), the hazard ratio for mortality associated with depression among RA patients was 1.38 (1.24–1.54; *p*-value <0.001). Regarding subtypes of depression, the average age at diagnosis of RA patients with major and minor depression was 53.43 and 56.26, respectively. The mean LE for major and minor depression was 26.70 and 23.33 years, respectively. The loss-of-LE was 4.68 years (1.56–10.64) for major depression and 5.46 years (1.64–9.19) for minor depression (refer to Table 1 and Figure 1). However, no statistically significant difference was observed (*p*-value = 0.822).

**Table 1:** Life expectancy, loss of life expectancy, lifetime cost, and cost per life-year of rheumatoid arthritis patients, stratified by depression.

	*N*	Death	Men	Age	LE (SEM)	Loss-of-LE (SEM)	Lifetime cost (SEM)	Cost per year
RA without depression	41,649	6,998 (16.8%)	9,807 (23.5%)	52.68 ± 14.11	27.05 (0.60)	4.76 (0.60)	92,239 (1,593)	3,812
RA with depression	1,662	344 (20.7%)	279 (16.8%)	55.41 ± 13.12	24.00 (1.83)	5.60 (1.85)	90,346 (5,207)	4,123
Major depression	500	101 (20.2%)	73 (14.6%)	53.43 ± 13.19	26.70 (2.34)	4.68 (2.40)	99,178 (7,314)	4,228
Minor depression	1,162	243 (20.9%)	206 (17.7%)	56.26 ± 13.12	23.33 (2.10)	5.46 (2.18)	87,109 (6,272)	4,061

**Figure 2:**
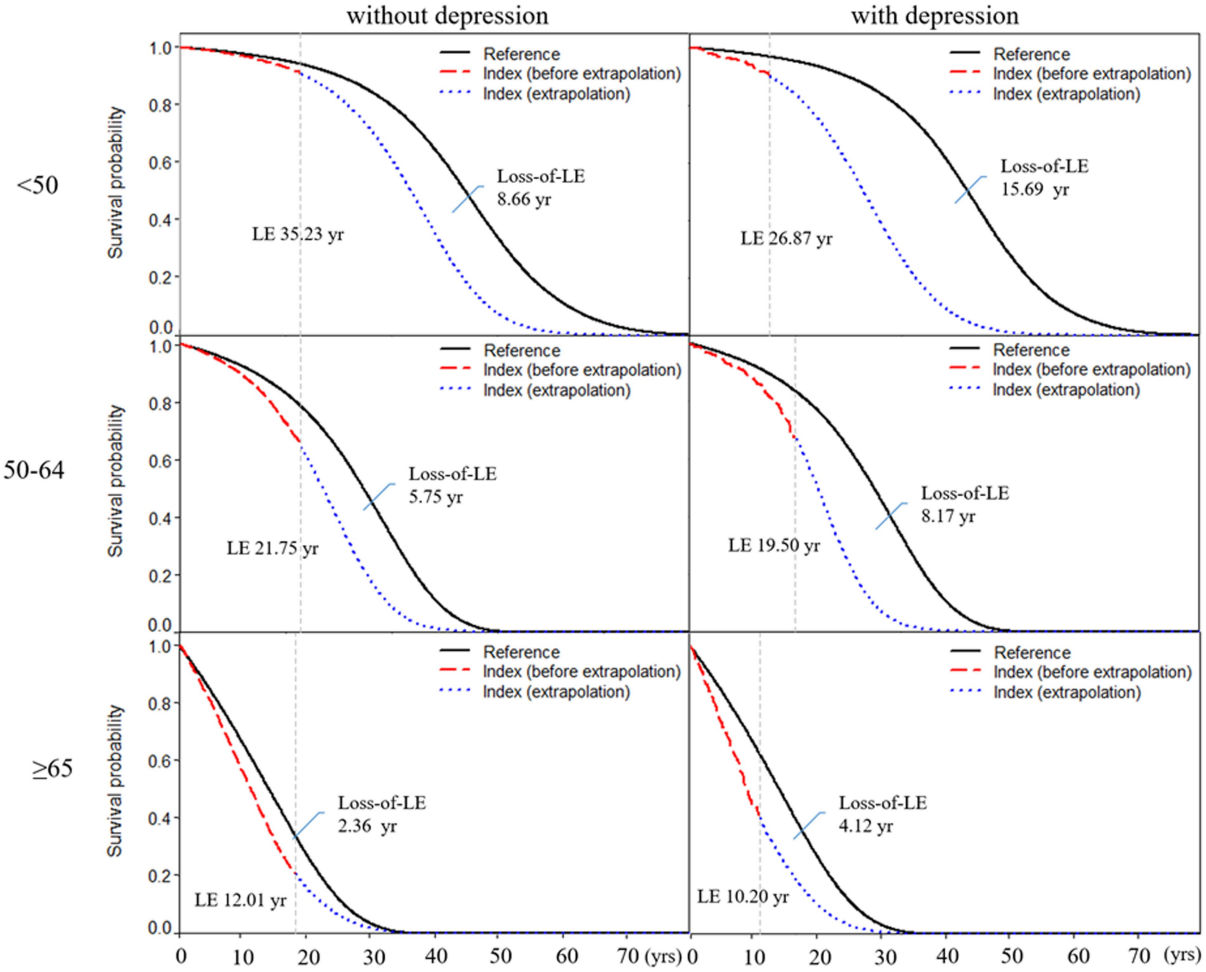
Life expectancy and loss of life expectancy after diagnosis of rheumatoid arthritis stratified by depression and age at diagnosis. The red line indicates the survival curve of true data from rheumatoid arthritis patients, and the blue line indicates the survival curve of extrapolation from rheumatoid arthritis. The black line indicates the survival curve of the sex-, age-, and calendar year-matched reference cohort.

**Figure 3:**
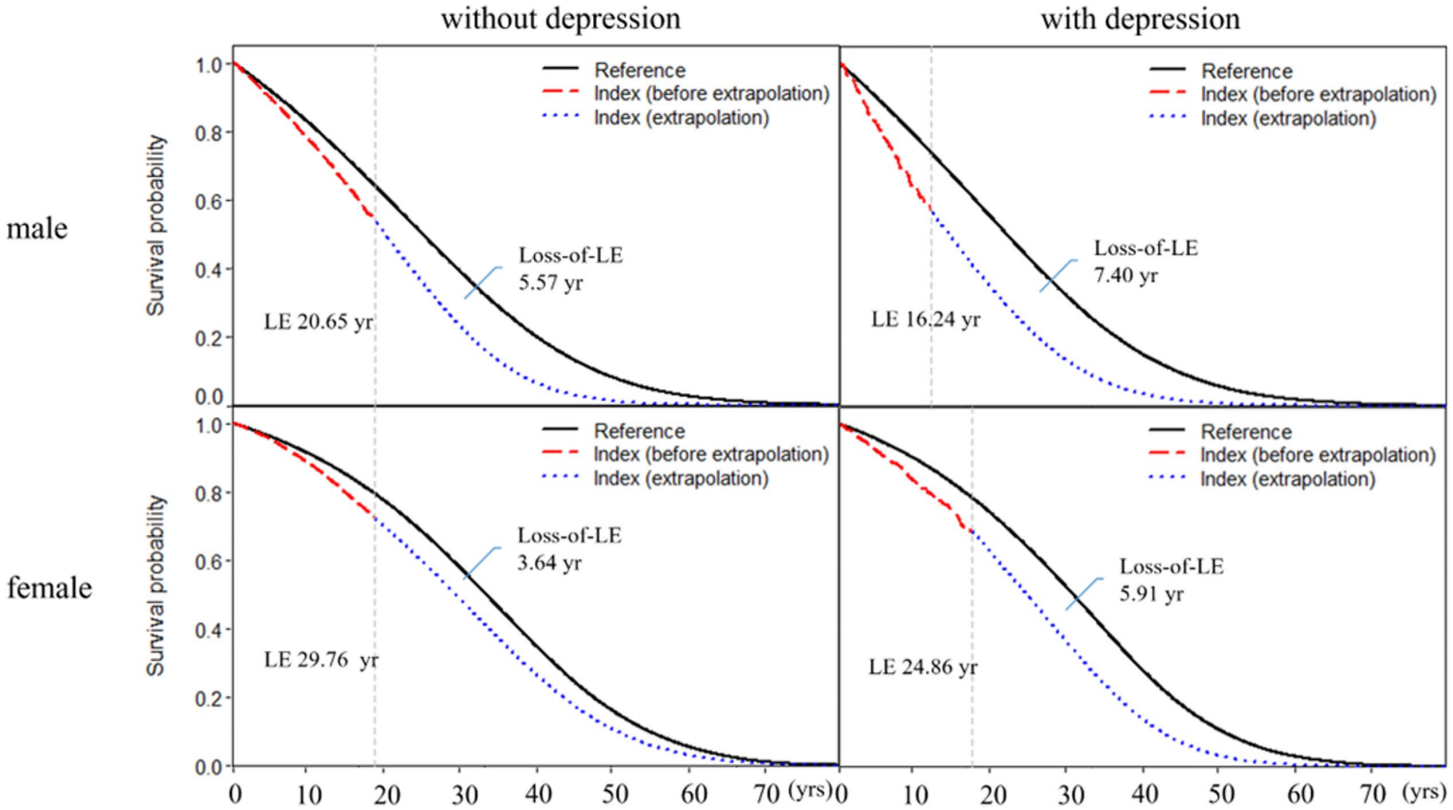
Life expectancy and loss of life expectancy after diagnosis of rheumatoid arthritis stratified by depression and sex. The red line indicates the survival curve of true data from rheumatoid arthritis patients, and the blue line indicates the survival curve of extrapolation from rheumatoid arthritis. The black line indicates the survival curve of the sex-, age-, and calendar year-matched reference cohort.

Table 2 shows subgroup analysis stratified by sex and age for RA patients with and without depression. We found that the loss-of-LE for male RA patients with and without depression was 7.40 years (2.14–11.4) and 5.57 years (4.70–11.0), respectively. Similarly, the loss-of-LE for female RA patients with and without depression was 5.91 years (1.53–9.93) and 3.64 years (2.31–5.65), respectively. These results indicate that, in our study, male patients had lower LE when compared to female patients. It is worth noting that the difference in loss-of-LE due to depression was not significant for both sexes. The loss-of-LE for RA patients without depression was 8.66 (2.88–12.16), 5.75 (4.77–6.34), and 2.36 (2.12–2.66) years for those aged <50, between 50 and 64, and ≥65, respectively. Among RA patients with depression, the loss-of-LE was 15.69 (2.86–21.83), 8.17 (4.42–9.89), and 4.12 (2.50–5.52) years for those aged <50, between 50 and 64, and ≥65, respectively. These findings indicate that study patients with RA onset at younger and middle-aged ages have significantly greater loss-of-LE than older-aged patients, particularly those with depression. In addition, when comparing the loss-of-LE between RA patients with and without depression, it was only statistically significant among those aged ≥65 years (*p*-value = 0.036). Figures 2, 3 indicate the correlation between LE and loss-of-LE in these stratifications.

**Table 2:** Life expectancy, loss of life expectancy, lifetime cost, and cost per life-year of rheumatoid arthritis patients, stratified by depression, sex, and age at diagnosis.

	Without depression							With depression						
Sex	*N*	Death, *n* (%)	Age, mean (SD)	LE (SEM)	Loss-of-LE (SEM)	Lifetime cost (SEM)	Cost per year	*N*	Death, *n* (%)	Age, mean (SD)	LE (SEM)	Loss-of-LE (SEM)	Lifetime cost (SEM)	Cost per year
Male	9,807	2,386 (24.3%)	55.33 ± 14.11	20.65 (0.76)	5.57 (0.77)	72,740 (2141)	3,741	279	96 (34.4%)	58.59 ± 13.89	16.24 (2.43)	7.40 (2.53)	71,348 (8,247)	4,594
Female	31,842	4,612 (14.5%)	51.87 ± 14.01	29.76 (0.93)	3.64 (0.94)	99,000 (2255)	3,813	1,383	248 (17.9%)	54.77 ± 12.97	24.86 (2.34)	5.91 (2.37)	94,053 (6,441)	4,138

Age	*N*	Death, *n* (%)	Men, *n* (%)	LE (SEM)	Loss-of-LE (SEM)	Lifetime cost (SEM)	Cost per year	*N*	Death, *n* (%)	Men, *n* (%)	LE (SEM)	Loss-of-LE (SEM)	Lifetime cost (SEM)	Cost per year
<50	17,651	755 (4.3%)	3,424 (19.4%)	35.23 (2.54)	8.66 (2.55)	112,891 (4,957)	3,678	566	40 (7.1%)	74 (13.1%)	26.87 (4.97)	15.69 (4.94)	100,721 (10,982)	4,038
50–64	15,641	2,315 (14.8%)	3,827 (24.5%)	21.75 (0.39)	5.75 (0.39)	86,265 (1,874)	4,116	702	116 (16.5%)	112 (16%)	19.50 (1.46)	8.17 (1.46)	81,751 (5,972)	4,283
≥65	8,357	3,928 (47%)	2,556 (30.6%)	12.01 (0.14)	2.36 (0.14)	48,823 (790)	4,100	394	188 (47.7%)	93 (23.6%)	10.20 (0.79)	4.12 (0.83)	52,242 (4,566)	5,144

### 3.3. Estimation of lifetime healthcare expenditures for patients

The lifetime costs of RA patients without depression and with depression were USD $92,239 and USD $90,346, respectively. Patients with RA without depression had higher lifetime costs than those with depression due to longer LE. However, to account for LE, we divided lifetime costs by LE to obtain the cost per year. The annual healthcare cost, derived from the division of lifetime healthcare costs by life expectancy, amounted to USD $4,123 for patients with both RA and depression and USD $3,812 for patients with RA alone (*p*-value = 0.043).

**Figure 4:**
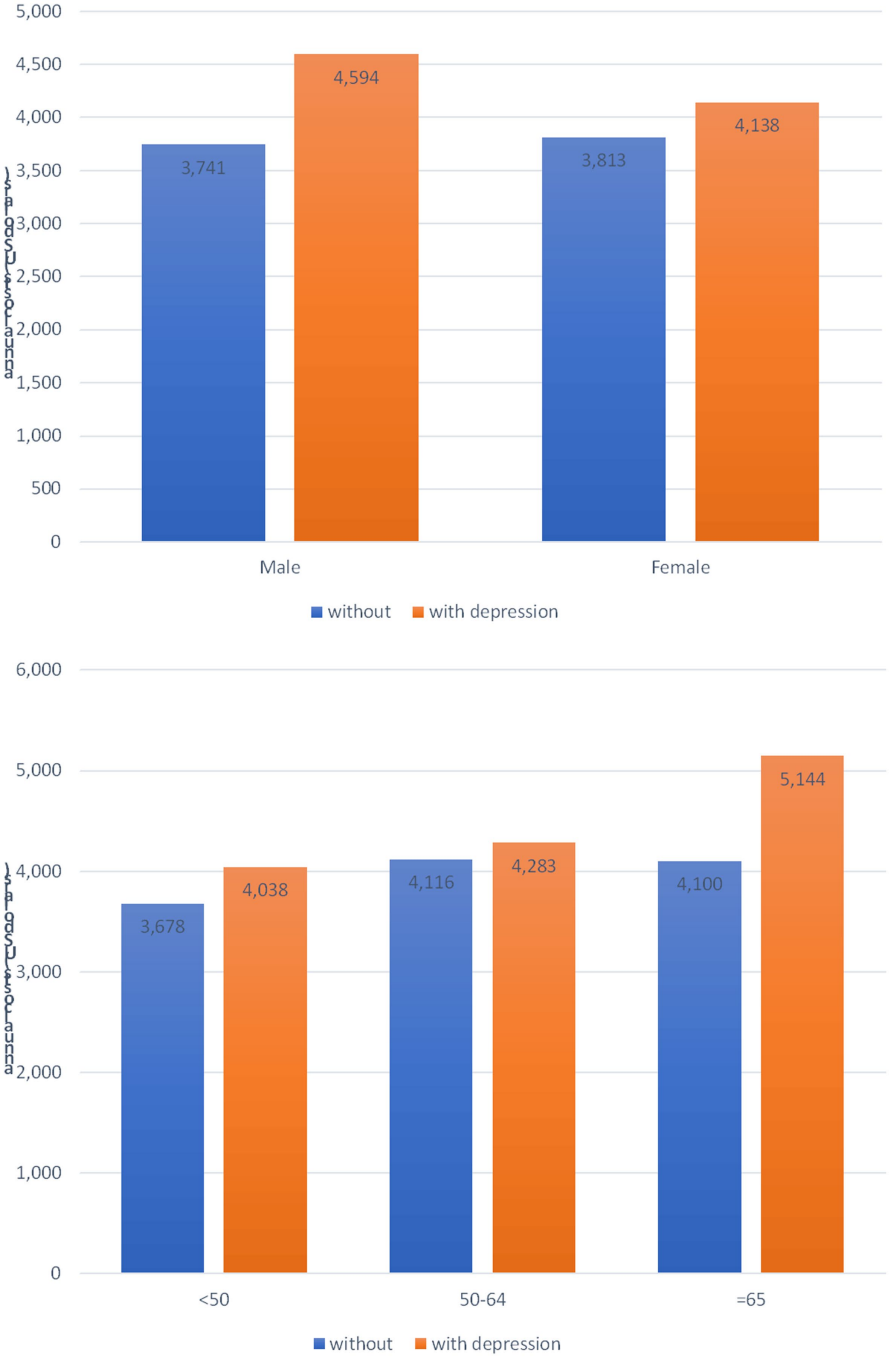
Annual healthcare cost after diagnosis of rheumatoid arthritis stratified by the sex and age groups.

Figure 4 illustrates the annual healthcare costs categorized by the sex and age groups. When comparing the annual healthcare costs between patients with and without depression, a disparity of USD $853 (*p*-value = 0.024) was observed among male patients and USD $325 (*p*-value = 0.046) among female patients. Regarding age groups, the difference amounted to USD $360 (*p*-value = 0.206) for individuals aged <50 years, USD $167 (*p*-value = 0.473) for those aged between 50 and 64 years, and USD $1,044 (*p*-value = 0.001) for those aged ≥65 years.

### 3.4. Sensitivity analysis

The findings from the sensitivity analysis, encompassing RA patients diagnosed from 2003 to 2017, remained consistent (see Table 3). A modest elevation in LE and a slight reduction in loss-of-LE were observed alongside a marginal increase in annual healthcare costs. It was noted that most of the initial study cohort was incorporated after introducing biological therapy. Consequently, the outcomes exhibited no significant alteration.

**Table 3:** Sensitivity analysis for life expectancy, loss of life expectancy, lifetime cost, and cost per life-year of rheumatoid arthritis patients diagnosed between 2003 and 2017.

	*N*	Death	Men	Age	LE (SEM)	Loss-of-LE (SEM)	Lifetime cost (SEM)	Cost per year
RA without depression	29,156	3,462 (11.8%)	7,224 (24.7%)	53.10 ± 14.18	27.84 (1.12)	3.63 (1.13)	90,854 (2,127)	3,852
RA with depression	1,251	209 (16.7%)	212 (16.9%)	55.85 ± 13.32	24.24 (2.52)	5.11 (2.58)	89,843 (6,367)	4,262

## 4. Discussion

To the best of our knowledge, this is the first study to estimate both LE and lifetime healthcare costs in a cohort of patients with incident RA with and without depression. We found that the loss-of-LE was 5.60 years for patients with RA with depression and 4.76 years for those with only RA. The annual healthcare costs were USD$ 4,123 for RA patients with depression and USD$ 3,812 for those without depression. In the subgroup analysis, the loss-of-LE for male patients with RA was larger than that for female patients, but the sex difference in loss-of-LE was similar across patients with and without depression. On the other hand, the annual medical costs were similar for both sexes among patients with RA alone but much higher for male RA patients with depression than for female RA patients with depression. Regarding group, the loss-of-LE was much higher among RA patients with depression aged <50 years; however, the annual medical costs were much higher among RA patients with depression aged ≥65 years.

In this study, the prevalence of depression among patients with RA was 3.8%, which was higher than that of the general population in Taiwan (22). However, the prevalence is much lower than the pooled prevalence (16.8%) estimated in one meta-analysis (4). It should be noted that the prevalence of depression among patients with RA varied across different countries and study designs. The prevalence of depression in the general population is approximately 1%–2% in Taiwan (23), which is much lower than that in other countries (24). Moreover, the prevalence of depression was relatively higher using self-reported instruments than that diagnosed by psychiatric interviews (4). A study using US claim records found that the prevalence was 7.5% among incident RA patients (14). Another study conducted in Japan found that the prevalence was 5% (15). Notably, the treatment rate also affects the estimated prevalence using the claim database. Overall, the treatment rate for depression varied, ranging from 16.8% in low-income countries to 48.3% in high-income countries (25). In Taiwan, the treatment rate was approximately 27% of patients (26).

Furthermore, we only identified pre-existing depression cases in the year preceding the RA diagnosis. Patients who developed depressive disorders after RA onset were not identified in our study but would be in a cross-sectional design. Thus, the prevalence of depression in the present study was relatively low. In our post-hoc analysis, we identified 5,316 individuals (12.8%) who developed depressive disorders among the comparison groups during the follow-up period. The incidence rate was calculated at 25.7 per 1,000 person-years.

We observed a greater loss-of-LE in patients with both RA and depression compared to those with RA alone, despite the overlapping 95% confidence intervals for the loss-of-LE in these two groups. Nevertheless, employing the Cox regression model, we identified a statistically significant hazard ratio for mortality associated with depression. This finding was consistent with previous studies that showed that RA patients with depression had high mortality (10, 11). The underlying mechanism is multifactorial. Although RA patients with depression might be more likely to die by suicide (12), increased mortality was not restricted to suicide. Patients with depressive disorder have unhealthy lifestyles, including a lack of exercise and an unhealthy diet (27), which could be attributed to non-suicide mortality. One study demonstrated that depression increased the risk of myocardial infarction among patients with RA (28). Furthermore, depression is associated with the inflammation process, which might exacerbate RA activity (29). Moreover, depression might be associated with a poor treatment response for RA, which further deteriorates the prognosis of RA (30, 31). All of these factors are associated with the increasing loss-of-LE among RA patients with depression.

Unsurprisingly, patients with RA and depression had higher annual healthcare costs than those with RA alone. In this study, we did not assess the disease-specific costs. Several studies found that patients with RA and depression had higher all-cause healthcare utilization and costs (13–15). One study demonstrated annual healthcare costs of USD$ 12,225 for RA patients with depression and USD$ 11,404 for those with RA only, with a 7% incremental cost (14). However, the percentage of incremental healthcare costs for depression was 46% in a Japanese study (15). In our study, the annual medical cost of depression increased by 8.2%, which was much closer to the findings in the US. The increased healthcare cost might be due to the cost of depression treatment; however, previous studies showed that RA-related healthcare utilization also increased (13–15). Comorbid depression might also be associated with poor RA treatment responses (30, 31). In addition, we only calculated the costs reimbursed in the NHI program, and this study did not include out-of-pocket expenses. Therefore, medical costs were underestimated. The components of increased healthcare costs and the underlying mechanisms should be explored in detail.

In terms of depression subtypes, the annual healthcare cost for major depression was higher than that for dysthymia or minor depression. However, we found that there was no difference in the loss-of-LE between major and minor depression. A previous study showed that the mortality risk for dysthymia was similar to that for major depression (32). Patients with dysthymia had fewer depression symptoms but lower rates of remission (33). Further research is needed to determine which symptom severity or illness duration determinants had a higher effect on LE among RA patients.

We found that older patients with RA had higher annual healthcare costs but less loss-of-LE. Older patients with depression might have more comorbidities and healthcare costs (34). Additionally, older patients have more risk factors for mortality; therefore, the effect of depression on mortality was less prominent than other risk factors. In contrast, depression may be among the few major risk factors among younger adults. Hence, depression had an important effect on mortality in our study.

The sex difference in loss-of-LE between patients with and without depression was similar; however, the annual medical costs of male patients with depression were higher than those of female patients. Whether there is a sex-related difference in the outcome of RA remains inconclusive (35). Possible factors associated with the outcome include hormonal factors, help-seeking behaviors, and medication compliance. The treatment rate for depression was lower in men than in women, and undertreatment might deteriorate treatment response and increase costs (26). No previous study has explored the role of sex on the association between depression and mortality or medical costs among patients with RA. Further investigations are necessary to explore these differences.

This study has several limitations. First, we identified patients with depressive disorders based on claim records. The prevalence of depressive disorders was found to be lower than in a previous study (4). According to our previous investigation, only 27% of patients sought treatment for depression (26). It is possible that some patients with depression who were neither diagnosed nor treated could have been misclassified into the comparison group. Conversely, while the accuracy of ICD codes for depressive disorders is substantial, some patients without depressive disorders might have been misclassified into the depression groups. These misclassifications could, overall, reduce the observed differences in LE and lifetime healthcare costs between RA patients with depression and those without depression. Therefore, our results may have been underestimated. Second, in order to avoid immortal time bias (36)—wherein RA patients must survive until depression diagnosis—we refrained from categorizing patients who developed depressive disorders after RA diagnosis into the depression groups. It is important to acknowledge that such misclassifications could potentially lead to underestimations in our findings. Third, we only measured direct healthcare costs. Quality of life, functional disability, loss of productivity, and caregiver load were not included. The economic burden of depression with RA is greater than the direct healthcare costs. Fourth, given the limited sample size, we did not explore the causes of death, and these findings should be explored in future. Fifth, we matched for age, sex, and calendar year. Nevertheless, certain variables, including socioeconomic status, disease severity, comorbid medical conditions, and the introduction of biological therapies throughout the study duration, could have significantly affected costs and outcomes, potentially confounding our findings. These variables may also act as mediators in the relationship between depression and lifetime healthcare costs in individuals with RA. Consequently, we refrained from making adjustments for these factors. Finally, we did not investigate the treatment effect of depression on the loss-of-LE and direct medical costs. Previous studies have shown that antidepressants might be associated with a reduced risk of diabetic complications among patients with diabetes (37). It should also be investigated whether depression treatment could alleviate the adverse effects of depression.

Despite the abovementioned limitations, this study’s strengths include the use of a nationally representative cohort with an 18 years follow-up period, a relatively large cohort size, and a novel method for estimating LE and lifetime healthcare expenditure.

## 5. Conclusion

We found that patients with RA had a higher loss-of-LE and higher direct medical costs when they had comorbid depressive disorders. Depression is a treatable illness but is commonly unrecognized and undertreated. Because the effect of depression is noticeable, clinicians should focus on screening for and detecting depression among patients with RA. Whether treatment can prolong LE and reduce healthcare costs remains unclear. Further research should focus on evaluating the effect of depression treatment on advanced complications and mortality in patients with RA.

## Data availability statement

The data analyzed in this study is subject to the following licenses/restrictions: data used in this study are from the Health and Welfare Data Science Center at Ministry of Health and Welfare, which are not publicly available. Requests to access these datasets should be directed to the Health and Welfare Data Science Center (HWDC), https://www.apre.mohw.gov.tw/.

## Ethics statement

The studies involving humans were approved by the Research Ethics Committee of China Medical University and Hospital (IRB number: CMUH108-REC2-119). The studies were conducted in accordance with the local legislation and institutional requirements. The ethics committee/institutional review board waived the requirement of written informed consent for participation from the participants or the participants’ legal guardians/next of kin because all personal information is de-identified.

## Author contributions

Y-MC, W-LH, and C-SW contributed to conceptualization, methodology, data curation, and original draft preparation. J-LL and W-LH supervised, reviewed, and edited the draft. All authors contributed to the article and approved the submitted version.
